# Contributions of Indian conservative dentists and endodontists to the *Medline database* during 1996–2009: A bibliometric analysis

**DOI:** 10.4103/0972-0707.73374

**Published:** 2010

**Authors:** Saravanan Poorni, S Ramachandran, T Rooban, PD Madan Kumar

**Affiliations:** Department of Conservative Dentistry and Endodontics, Ragas Dental College and Hospital, Uthandi, Chennai 600 119, India; 1 Department of Oral and Maxillofacial Pathology, Ragas Dental College and Hospital, Uthandi, Chennai 600 119, India; 2 Department of Public Health Dentistry, Ragas Dental College and Hospital, Uthandi, Chennai 600 119, India

**Keywords:** Conservative dentists and endodontists in India, conservative dentists and endodontists’ publications, publication trends

## Abstract

**Background:**

Analysis of publication trends will guide the policy framers, administrators, and dentists to frame future policies and design programs for the development of scientific and technological policies in the field of dentistry.

**Aims and Objectives:**

This study was undertaken to assess the trends in Indian Conservative dentists and endodontists’ Publication in PubMed-Medline database during 1996–2009.

**Materials and Methods:**

Using the time limitation of publication date limit of 1^st^ January 1996 to 31^st^ December 2009, all articles where authors’ affiliation had the words Dental AND India were selected. From this collection of articles, the following were noted down: year of publication, number of authors, name of the journal, reach of the journal, status of the journal, specialty of the first, state of origin, and type of research. From this database, the performance of department of conservative dentistry and endodontics was analyzed.

**Results:**

The number of articles published by conservative dentists and endodontists was 124. Among them, 63 got published in international journals and 61 in Indian journals. A majority of 33 journals were published in *Indian Journal of Dental Research* followed by 25 in the *Journal of Conservative Dentistry*. Out of these articles, 66 were on the basis of original research done by the authors. Nearly 45.2% of the published articles were from the institutes in Tamil Nadu, followed by Karnataka (30.6%), and Maharashtra (8.1%). Although the overall distribution of the publication trends seems to be constant from 1996 to 2006, there seems to be boom in the publication trend since 2007.

## INTRODUCTION

The health sciences are undergoing great changes in the way they produce, use, and interpret knowledge; hence professionals must constantly try to keep abreast of the latest advances. The analysis of scientific research in biomedical fields is a complex process, and no methodology has been developed that fully satisfies the needs of researchers, institutions, and administrators. The analysis of publications is one of the most widespread approaches, but has also drawn the most criticism. Despite the controversies, very few objective data have been published to determine research ranking in the field of dentistry. This kind of analysis may help inform the development of scientific and technological policies in dentistry, of special relevance in emerging economies that are currently undergoing rapid transformation.[1]

India had ranked 26^th^ position in terms of number of peer-reviewed published dental manuscripts in the period 1999–2003 using ISI database approach (0.66% of all contribution globally). This was produced by 366 authors, with a productivity of 0.361, a mean of inter-annual variation rate of percentual average increase during the study of the productivity and the number Indian authors as 16.31, relative specialization index as 11.01, specific weighting of the dental scientific production of India in relation to its general scientific production as 0.241, weighted impact factor of 1.025 and relative impact factor of 0.964, mean citation rate for each document as 2.05 and nine documents from India in the five top 5 journals with highest impact factor.[1]

PubMed-Medline is a widely used database created by the National Center for Biotechnology Information and National Institute of Health, USA. This database is updated almost daily and contains details of millions of manuscripts in the field of life sciences. It is the most widely used search tool for millions of health and life sciences researchers. PubMed-Medline is the NLM’s (National Library of Medicine, USA) premier online bibliographic database which is freely accessible, and covers all the fields such as medicine, nursing, dentistry, veterinary medicine, health care system, and the preclinical sciences.[2]

Against this background of achievement and ongoing challenges, this study was undertaken to assess the trends in Conservative Dentistry and Endodontics Publication by Indians in PubMed-Medline database during 1996–2009.

## MATERIALS AND METHODS

During the first fortnight of January 2010, data were collected using the method as described below. Using the time limitation of publication date limit of 1^st^ January 1996 to 31^st^ December 2009, all articles where author’s affiliation had the words “Dental” AND “India” were selected. No journal or types of article limitation were set. All articles, from all types of journal including basic sciences, clinical medical sciences, and dental journals were included. All the articles that were displayed were considered for the analysis. The following criteria were followed for accumulating the data: (A) First author’s affiliation was only considered for the study. (B) Non-dental (medical articles) from dental institutions were also included for the study, but under a separate category. (C) Only Indian institutions were considered for the study.

From this collection of articles, the following were noted down: year of publication, number of authors, name of the journal, reach of the journal (depending on the readership–specialty/general dental/medical journal or others), status of the journal (published in India or at other nations), specialty (dental) of the first author (all dental specialties, basic medical sciences, or others), state of origin, type of research (case reports [including case series]/reviews [excluding systematic review]/original research [encompass epidemiological, comparative, qualitative, cross sectional, longitudinal, *In vivo* and clinical trial studies]/technical notes/others [such as editorial, *etc*]). There were certain issues such as improper citation of name of the institution/department and noncitation of state of origin. Considering the data as visible on the National Institute of Health Website, the database for the study was prepared and no corrections and addition/deletions were performed by the author. Those data that were missing were labeled as “not mentioned”.

All data thus gathered were entered in SPSS software version 16.0. From this database, the performance of Department of Conservative Dentist and Endodontist was presented. Descriptive analysis was performed and presented. Interannual distributions, statewise distribution, and types of research are presented. Mean number of authors per document in each year is also presented. Microsoft Excel 2007 was used to assess the trend analysis using the present trend of growth of research outputs.

## RESULTS

Employing the methodology mentioned, search was carried out in first fortnight of January 2010. The number of articles published by Conservative Dentists and Endodontists was 124. Among them, 63 got published in international journals and 61 in Indian journals.Table 1 shows the list of journals which had publications by Conservative Dentists and Endodontists during the study period. A majority of 33 journals were published in *Indian Journal of Dental Research* followed by 25 in the *Journal of Conservative Dentistry*. Table 2 shows the distribution of publications by Conservative Dentists and Endodontists, along with the mean number of authors, when the journals were categorized based on their type, location of publication, and the type of articles published. Out of the overall 124 articles, 66 were on the basis of original research done by the authors. Table 3 shows the statewise distribution of the publication trends during the study period. Nearly, 45.2% of the published articles were from the institutes in Tamil Nadu, followed by Karnataka (30.6%) and Maharashtra (8.1%). Table 4 shows the trend of publications by Conservative Dentists and Endodontists during the study period. Although the overall distribution of the publication trend seems to be constant from 1996 to 2006, there seems to be boom in the publication trend in 2007 with around 21 articles in 2007 that has increased to 41 in 2009.

**Table 1:** List of journals which had publications by Conservative Dentists and Endodontists during the study period (1996–2009)

Name of Journal	Number of articles published
*Aust Endo J*	3 (2.4)
*Am J Ortho Dentofacial Orthopaedics*	1 (0.8)
*California JCDA*	1 (0.8)
*Dental Traumatology*	2 (1.6)
*Dental Update*	2 (1.6)
*IEJ*	2 (1.6)
*Indian J Dent Res*	33 (26.6)
*Int Endod J*	1 (0.8)
*Int J Pedia Dent*	1 (0.8)
*J Clin Pedia Dent*	2 (1.6)
*J Cons Dent*	25 (20.2)
*J Endo*	21 (16.93)
*J Indian Soc Pedod Prev Dent*	3 (2.4)
*J Cont Dent Practice*	1 (0.8)
*J Oral Rehabil*	1 (0.8)
*Kathmandu Univ Med J (KUMJ)*	7 (5.6)
*N Y State Dent J*	2 (1.6)
*Oper Dent*	6 (4.8)
*Oral Surg Oral Med Oral pathol OralRadiol*	9 (7.2)
*Endod*	
*Quintessence Int*	1 (0.8)
*Total*	124

Figures in parentheses are in percentage

**Table 2:** Depicting the mean number of authors among reach, status of journals, and type of articles

Type of journal based on reach	Number of publications	Mean ± SD number of authors	*P* value
Type of journal based on reach			0.285
Specialty dental journals	69 (55.6)	3.12 ± 1.34	
General dental journals	46 (37.1)	2.89 ± 1.15	
Medical journals	7 (5.6)	3.86 ± 0.69	
Others	2 (1.6)	3	
Type of journal based on location of publication			0.177
Indian	61 (49.2)	2.92 ±1.38	
International	63 (50.8)	3.22 ± 1.09	
Type of article			0.00
Case report	35 (28.2)	3.57±1.19	
Research	66 (53.2)	3.11± 1.15	
Technical note	1 (0.8)	2	
Review	18 (14.5)	2.5 ± 1.2	
Editorial	4 (3.2)	1	

Figures in parentheses are in percentage

**Table 3:** The statewise distribution of the publication trends during the study period

Name of the state	Number of publications	Mean ± SD number of authors
Karnataka	38 (30.6)	3.21 ± 1.31
Tamil Nadu	56 (45.2)	3.18
Punjab	2 (1.6)	1.5 ± 0.7
Maharashtra	10 (8.1)	3.7 ± 1.63
Goa	1 (0.8)	3
Gujarat	1 (0.8)	2
Haryana	8 (6.5)	2.38 ± 0.51
Madhya Pradesh	1 (0.8)	3
Pondycherry	2 (1.6)	1
Rajasthan	1 (0.8)	2
Uttar Pradesh	4 (3.2)	2.5 ± 0.57
Total	124	

Figures in parentheses are in percentage

**Table 4:** Distribution based on the trend of publications by Conservative Dentists and Endodontists during the study period

Year of publication	Number of publications
1996	1 (0.8)
1997	1 (0.8)
1998	4 (3.2)
1999	5 (4)
2000	3 (2.4)
2001	3 (2.4)
2002	5 (4)
2003	4 (3.2)
2004	2 (1.6)
2005	6 (4.8)
2006	9 (7.3)
2007	21 (16.9)
2008	19 (15.3)
2009	41 (33.1)
Total	124

Figures in parentheses are in percentage

**Figure 1:**
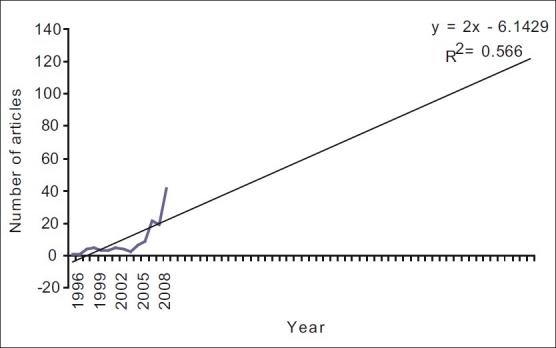
Trend analysis using 1996-2009 data

**Figure 2:**
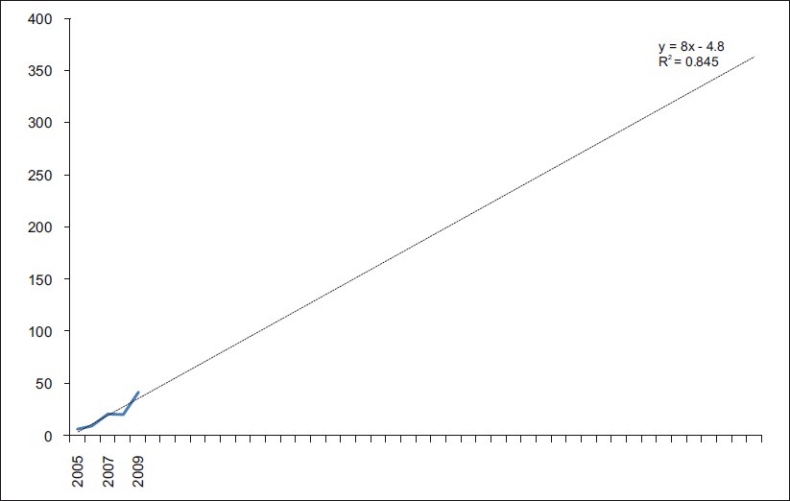
Trend analysis using 2005-2009 data

The projection of the growth trend is depicted in Figures 1 and 2. By the year 2050, approximately 108 articles are expected to be produced if the trend of 1996–2007 continues. However, if the trend of 2005–2009 were considered, the number of article per year in 2050 would be above 360.

## DISCUSSION

Health care professionals usually base their decisions on professional experience, prevailing clinical practice, standard procedures, training or expert guidance, peer consultation, and knowledge acquired in dental school, seminars, and continuing education programs. Clinical experience, technical expertise, and critical judgment are essential, but not sufficient. Due to the complexity of information, scientific support to clinical practice must be sought in the medical literature and derived from methodologically validated tools. Therefore, it is essential that surveys should be undertaken, for they provide scientific basis to professionals in their search for better quality of life standards for the population. Hence, the importance of bibliometrics, which is being increasingly used in analyses of scientific production, and has become a statistical support device that allows mapping and generating different information and knowledge handling and management indicators, particularly in scientific, technological, and productivity-related information and communication systems necessary to the planning, evaluation, and management of a given scientific community or country.[3]

Publication analysis, as with any method chosen to assess scientific production, does not cover the entirety of scientific production. However, PubMed-Medline is not representative of entirety of the peer-reviewed publication.[1] However, similar approach has been successfully used in other partial analysis of dental research production.[2,4–6] Furthermore, this article suffers from a drawback of being quantitative and not qualitative.

Most of these articles published by Conservative Dentists and Endodontists are from institutional and largely private institutions. There is a drastic increase in the number of publications since 2007. This phenomenon could be explained by an increase in the number of Dental colleges across India and conservative dentistry and endodontics post-graduation seats.[7] As there are very few articles in this aspect to refute the results of this study, the results of this study cannot be compared against any published literature.

Of late, there has been an encouraging trend in the *Journal of Conservative Dentistry* (JCD), the official *Journal of Federation of Operative Dentistry*. The endeavor of the *Journal of Conservative Dentistry* is not only to provide a platform for the dissemination of knowledge being generated in this field across the country, but also to be the harbinger of change and betterment in clinical practice among professionals.[8] The mission of a journal is not only to serve as a portal of dissemination of research knowledge, but also to ensure that vital information reaches as many students, teachers, and researchers as possible. Indexing of a journal by reputed agencies helps one to achieve this task.[9] The indexing of *JCD* in Medline will definitely bring a welcome change in further increasing the publication trends among the Indian Conservative Dentists and Endodontists as they now have an indexed portal for reaching out their scientific contributions.

The drastic increase in publication in 2007 could also be due to the following reasons: (1) increase in quantity and quality of conservative dentists and endodontists, (2) change in policy of the Indian dental academic circles, (3) increasing opportunities, (4) increase in number of journals published from India, (5) increasing the global presence of Indian diasporas who helped their Indian dental friends, (6) increasing government funding opportunities or a combination of all or some of these factors. This is highlighted by the difference in trend analysis between Figures 1 and 2. This appreciation of numbers is a highly needed change to increase the global presence of Indian Conservative Dentists and Endodontists.

## CONCLUSION

Descriptive study of Conservative Dentists and Endodontists’ publication during 1996–2009 in PubMed-Medline database is presented. The loco-regional variation and interannual variation are presented. The result of this study clearly shows the lacunae of Conservative Dentists and Endodontists’ contribution to Indian Research Publications. Efforts should be made by Conservative Dentists and Endodontists in India to increase their global presence in the front of scientific contributions, as this information could be used by various professional societies, individual scientists, scholarly institutions, and funding organizations to frame essential policies regarding the improvement of the science of Conservative Dentists and Endodontists and to the benefit of common Indians.
